# A Beta Version of an Application Based on Computer Vision for the Assessment of Knee Valgus Angle: A Validity and Reliability Study

**DOI:** 10.3390/healthcare11091258

**Published:** 2023-04-28

**Authors:** Luis Ceballos-Laita, Xavier Marimon, Albert Masip-Alvarez, Sara Cabanillas-Barea, Sandra Jiménez-del-Barrio, Andoni Carrasco-Uribarren

**Affiliations:** 1 Department of Surgery, Ophthalmology, Otorhinolaryngology and Physical Therapy, Faculty of Health Sciences, University of Valladolid, 42004 Soria, Spain; luis.ceballos@uva.es (L.C.-L.);; 2 Bioengineering Institute of Technology, Universitat Internacional de Catalunya (UIC), 08195 Barcelona, Spain; 3 Automatic Control Department, Universitat Politècnica de Catalunya (UPC-BarcelonaTECH), 08034 Barcelona, Spain; 4 Institut de Recerca Sant Joan de Déu (IRSJD), 08950 Barcelona, Spain; 5 Department of Physiotherapy, Faculty of Medicine and Health Sciences, Universitat Internacional de Catalunya (UIC), C/Josep Trueta s/n, 08195 Sant Cugat del Vallès, Spain

**Keywords:** athletes, kinematics, computer-vision, validity study

## Abstract

Background: In handball, the kinematics of the frontal plane seem to be one of the most important factors for the development of lower limb injuries. The knee valgus angle is a fundamental axis for injury prevention and is usually measured with 2D systems such as Kinovea software (Version 0.9.4.). Technological advances such as computer vision have the potential to revolutionize sports medicine. However, the validity and reliability of computer vision must be evaluated before using it in clinical practice. The aim of this study was to analyze the test-retest and inter-rater reliability and the concurrent validity of a beta version app based on computer vision for the measurement of knee valgus angle in elite handball athletes. Methods: The knee valgus angle of 42 elite handball athletes was measured. A frontal photo during a single-leg squat was taken, and two examiners measured the angle by the beta application based on computer vision at baseline and at one-week follow-up to calculate the test-retest and inter-rater reliability. A third examiner assessed the knee valgus angle using 2D Kinovea software to calculate the concurrent validity. Results: The knee valgus angle in the elite handball athletes was 158.54 ± 5.22°. The test-retest reliability for both examiners was excellent, showing an Intraclass Correlation Coefficient (ICC) of 0.859–0.933. The inter-rater reliability showed a moderate ICC: 0.658 (0.354–0.819). The standard error of the measurement with the app was stated between 1.69° and 3.50°, and the minimum detectable change was stated between 4.68° and 9.70°. The concurrent validity was strong r = 0.931; *p* < 0.001. Conclusions: The computer-based smartphone app showed an excellent test-retest and inter-rater reliability and a strong concurrent validity compared to Kinovea software for the measurement of the knee valgus angle.

## 1. Introduction

Evaluating lower limb motion patterns in elite athletes is critical for understanding and analyzing sport-related movements. It enables healthcare professionals to identify and quantify these patterns, which can aid in injury prevention and the treatment of various dysfunctions. Handball, a sport that involves frequent jumps, landings, and changes of direction, requires a comprehensive study of lower limb kinematics [1,2]. Research indicates that 54% of handball injuries occur in the hip or knee region, with hamstring muscle strain and anterior cruciate ligament injury being the most common [3].

One of the leading contributing factors to knee injuries in athletes is the knee valgus angle during sport-related movements [4]. However, relying on visual observation alone to analyze the knee valgus angle is often subjective and prone to error [5]. Fortunately, the technological advancements in recent years allow clinicians to objectively quantify the knee valgus angle, leading to more accurate and reliable analysis [6]. By using these technologies, healthcare professionals can improve injury prevention strategies and develop effective treatment plans for athletes [7].

The valgus angle, which refers to the angle between the distal femur and tibia concerning the body’s midline, is a crucial measurement in understanding knee joint mechanics, as defined by Hewett et al. [8]. While advanced three-dimensional (3D) motion capture systems have traditionally been the gold standard for evaluating the knee valgus angle [9], they can be expensive and time-consuming to set up, making them impractical for many clinicians in daily practice [10]. As an alternative, the evaluation of the lower limb kinematics using two-dimensional (2D) software has been extensively explored [10,11,12,13,14,15]. Kinovea, a popular 2D motion analysis software, has emerged as a reliable tool for measuring lower limb kinematics in both frontal [14] and sagittal [13] planes. Balsalobre-Fernández et al. showed that Kinovea produces highly reliable values and can achieve excellent inter- and intra-rater reliability, even without prior experience [15]. While 2D analysis may not offer the same level of accuracy as 3D motion capture, it provides a more accessible and cost-effective solution for many clinicians. With the advent of technology and software such as Kinovea, the assessment of the valgus angle from 2D analysis is becoming increasingly popular and reliable.

The integration of computer vision in healthcare and sports medicine has the potential to revolutionize how patients and athletes are assessed and treated [16]. Previous studies analyzed recent technological advances, such as computer vision or deep learning, to analyze and extract data from digital images to track static and dynamic patterns [17,18]. This technology has demonstrated the ability to make reliable and valid records in humans and animals. In this study, the focus is on the assessment of the knee valgus angle using computer vision. The use of this technology may help clinicians and coaches to obtain more objective, accurate, and efficient measurements of the knee joint. This, in turn, can lead to better patient outcomes and improved performance in elite athletes. It seems that the use of computer vision and deep learning in healthcare and sports medicine is rapidly advancing, and its potential applications are vast. With the ability to track and analyze movement patterns, computer vision-based tools can provide valuable insights into injury prevention, rehabilitation, and performance optimization [19]. As technology continues to advance, it is likely that computer vision will become an increasingly prevalent tool in clinical practice, ultimately improving patient outcomes and enhancing athletic performance.

Ensuring the validity and reliability of data obtained from computer vision-based applications is of utmost importance for their effective use in clinical practice [19]. While a previous study has shown that computer vision-based tools can accurately measure joint angles during specific exercises such as the single-legged squat task [20], there is still a lack of research on the concurrent validity and reliability of such applications for measuring the knee valgus angle in elite athletes. In this regard, developing an application based on computer vision that can automatically evaluate the knee valgus angle can be immensely beneficial for healthcare providers and researchers [19,20]. With the help of such an application, clinicians can obtain a more precise and objective measurement of the knee joint angle, which can aid in identifying and treating injuries as well as improving the performance of elite athletes.

However, it is crucial to note that the validity and reliability of data generated by these novel applications based on computer vision must be thoroughly evaluated before they can be used in clinical practice. Hence, the aim of our study is twofold. Firstly, we aim to assess the test-retest and inter-rater reliability of the novel beta application based on computer vision for evaluating the knee valgus angle in elite high-performance athletes. Secondly, we aim to determine the concurrent validity of the beta application by comparing it with the 2D Kinovea software. This study seeks to contribute to the existing literature on the validity and reliability of computer vision applications for measuring joint angles, particularly in elite athletes. By establishing the accuracy and consistency of these tools, we can pave the way for their wider use in clinical practice, ultimately improving patient outcomes and enhancing the performance of athletes [15,19,20,21,22].

## 2. Materials and Methods

### 2.1. Study Design

A study on reliability and validity was developed in accordance with the Guidelines for Reporting Reliability and Agreement Studies [23]. The research was approved by the Ethical Research Committee of the University of Valladolid and registered under the number CASVE-NM-21-504B. The study also adhered to the ethical guidelines for clinical research involving human subjects as stated in the Helsinki Declaration.

The inclusion criteria for this study were male athletes practicing handball between the ages of 18 and 30 with a minimum of two years of experience as elite athletes and a practice routine of at least two hours per day and three days per week. Exclusion criteria were applied to those who presented with low back pain or pain in any joint of the lower limbs, a history of fracture, dislocation, or previous surgery in the lumbar spine or any joint of the lower limbs, neurological or musculoskeletal disorders, use of analgesics or muscle relaxants, and prior physiotherapy treatment within the last month on the lumbar spine or any of the lower limbs.

To recruit participants, two professional handball clubs from the second Spanish national league were contacted, and ultimately, 42 elite male handball athletes were enrolled in the study.

### 2.2. Sample Size

The sample size was calculated using the web-based sample size calculator for reliability studies developed by Arifin et al. A sample size of 42 participants was estimated with an α value of 0.05, a statistical power (1-β) of 80%, a number of measurements/examiners equal to 2, a minimum acceptable reliability of 0.75, and a 15% rate of dropouts [24].

### 2.3. Outcome Measurement

The knee valgus angle was measured using the beta application based on computer vision and the Kinovea software. The knee valgus angle was calculated as the angle formed between the line that joins the anterior superior iliac spine and the midpoint of the patella and in the midpoint of the patella and the anterior tibial tuberosity [25]. In addition, sociodemographic data such as sex, age, height, and weight were recorded.

#### 2.3.1. Knee Valgus Angle Measurement with the Beta Application Based on Computer Vision

**Figure 1:**
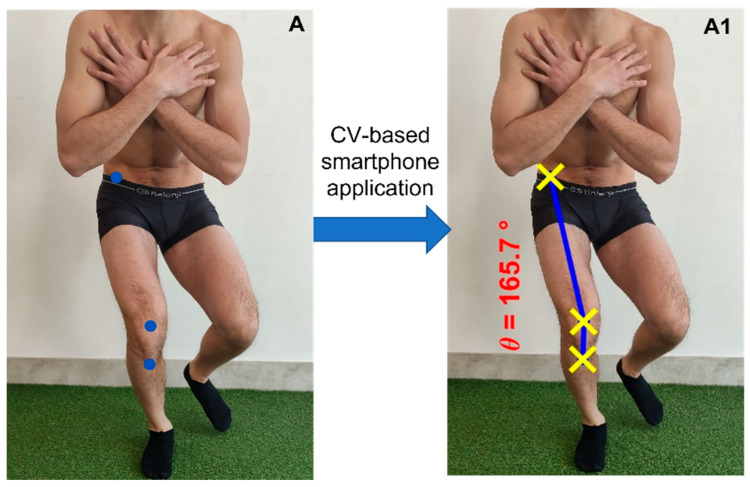
(**A**) Markers placed by the examiner. (**A1**) Knee valgus angle measured by the beta application based on computer vision.

For the assessment of the knee valgus angle, the beta application based on computer vision was used. This beta application works by detecting the markers on the photo. After detecting the markers, the application draws the lines needed to form the knee valgus angle and finally calculates the angle. The photo was taken using an iPhone (5SE). The phone was placed on a tripod perpendicular to the frontal plane. Between the tripod and the place where the handball athletes performed the single-leg squat was calculated a distance of 2 m, and the height of tripod was 1.05 m [26]. To calculate the knee valgus angle with the application, each examiner had to place the markers on the anterior superior iliac spine, midpoint of the patella, and the anterior tibial tuberosity. The markers had to have a diameter of 1.5 mm and had to be colored red or blue. Once the markers were placed, each examiner run the application with MATLAB and asked to the application to look for the photos and to evaluate the knee valgus angle of each participant (Figure 1). This beta application has shown strong current validity and excellent reliability in measuring the craniovertebral angle [17].

#### 2.3.2. Knee Valgus Angle Measurement with the Kinovea Software

The knee valgus angle was measured using the Kinovea software to analyze the concurrent validity comparing this instrument and the beta application based on computer vision. Kinovea has been previously used in several sports fields such as cycling, soccer, handball, and athletes. Kinovea has shown to be a valid instrument compared to AutoCAD, and its reliability was excellent. Several authors measured the reliability of Kinovea by assessing the lower limb kinematics in frontal and sagittal planes, which showed excellent values. Thus, Kinovea seems to be a free, portable, and easy-to-use software that may be used by clinicians and researchers [10,12,13,14,15,27].

The measurement by the Kinovea software was performed step-by-step by hand. The camera, the tripod, and the patient were placed in the same place described above. After taking the photo, the examiner had to measure the angle manually using the “angles” function [26].

### 2.4. Procedure

Before the training session, the handball athletes underwent an assessment in which their sociodemographic data, including age, height, weight, and body mass index, were recorded. Forty-two male elite handball athletes were included in the study. The participants included presented a mean age of 25.73 ± 6.18, a mean height of 189.23 ± 10.08 cm, a mean weight of 82.70 ± 14.34 kg, and a mean body mass index of 23.09 ± 3.15 kg/cm^2^.

After recording the sociodemographic data, a frontal photo was taken while each participant performed a single-leg squat.

To ensure consistency, the photos of each participant were taken in the same room prior to the training session. The environmental conditions, such as lighting and temperature, were maintained at a stable level throughout the process. Participants were instructed to perform a single-leg squat with their dominant leg, while the non-dominant leg was flexed at approximately 90°. Before the photo was taken, participants were allowed three practice attempts to familiarize themselves with the task [28].

**Figure 2:**
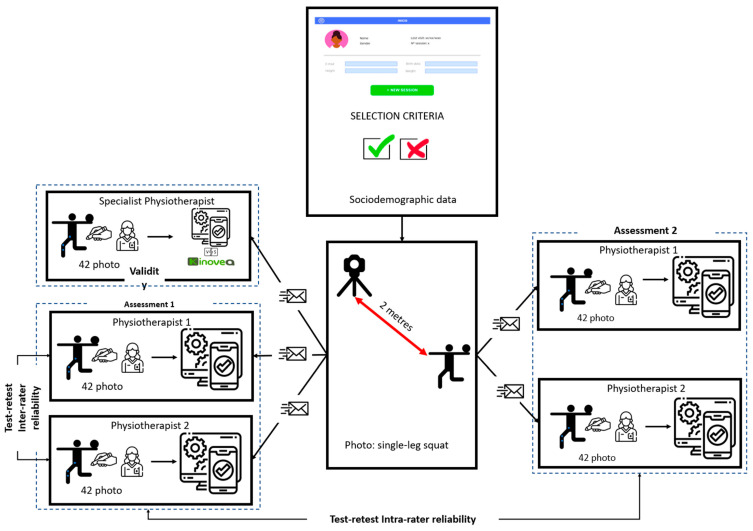
Procedure of the study.

To assess the test-retest reliability, each examiner assessed twice the same photo with a period of one week between assessments. Both examiners were blinded to the assessments of the other examiner. To check the concurrent validity of the beta application based on computer vision, a third examiner assessed the knee valgus angle of each photo using the Kinovea software version 0.9.4 (Available online: http://www.kinovea.org (accessed on 13 January 2023)) (Figure 2).

The time spent on each measurement was also recorded using the calculators of the instruments themselves.

### 2.5. Statistical Analysis

For data analysis, the study utilized the 20.0 version of the Statistical Package for the Social Sciences software for Windows (IBM, Chicago, IL, USA). Descriptive analysis of the quantitative variables was performed, with means (M) and standard deviations (SDs) calculated. Qualitative variables were analyzed using frequency and percentage calculations. To assess the reliability of the knee valgus angle measurement using the beta application based on computer vision, the study calculated the test-retest and inter-rater reliability using the ICCs and the 95% confidence interval (CI). The criteria to interpret the reliability of the results were as follows: ICCs below 0.50 indicated poor reliability, ICCs ranging from 0.50 to 0.75 indicated moderate reliability, ICCs ranging from 0.75 indicated good reliability, and ICCs above 0.9 indicated excellent reliability [29]. Additionally, the study calculated the standard error (SEM) and the minimum detectable change (MDC). The SEM was determined using the formula SD × √1 − ICC, while the calculation of the minimum level of detectable change (MDC95%CI) was based on the formula SEM × z-score at the two-sided 95% confidence intervals (z = 1.96) × √2 × SEM [29]. Finally, the coefficient of variation (CV) was calculated using the SD and the M of the repeated measurements; SD was divided by M and multiplied by 100 [30].

In order to assess the strength of the relationship between the assessment of the knee valgus angle using Kinovea software and the vision-based smartphone application, the study calculated the Pearson correlation coefficient. The strength of the correlation was interpreted as strong if it was greater than 0.70, moderate if it fell between 0.50–0.70, and low if it was less than 0.30 [31]. To further evaluate the agreement between the two methods, Bland–Altman plots were created using Microsoft Excel 2019 (17.0). These plots allow for the graphic comparison of between-instrument measurement error and the evaluation of any systematic bias. Specifically, the plots consist of differences between measurements on the *y*-axis and the mean values of the two instruments on the *x*-axis.

These analyses provide important information regarding the relationship and agreement between the two measurement methods, which is important for ensuring that the results obtained are both accurate and reliable.

## 3. Results

### 3.1. Test-Retest Reliability

Test-retest reliability was calculated by both examiners to ensure the consistency and stability of the results obtained. In particular, the knee valgus angle with the beta application was evaluated by the two examiners at baseline and after one week. The ICC values showed good reliability for Examiner 1, achieving an ICC of 0.859, and excellent reliability for Examiner 2, achieving an ICC of 0.933. These high ICC values indicate a high degree of agreement between the two examiners.

Table 1 shows the mean values, SDs, ICCs with 95%CI, SEMs, and MDCs for the knee valgus angle with the beta application based on computer vision assessed by both examiners. These measures provide important information about the reliability and precision of the measurement method. In particular, the SEM, MDC, and CV values indicate the degree of measurement error, the minimum detectable change, and the coefficient of variation, respectively.

### 3.2. Inter-Rater Reliability

In order to assess the consistency of the results obtained by two different examiners, the inter-rater reliability was calculated by comparing the data collected by Examiner 1 and Examiner 2 at baseline. The results showed a moderate ICC of 0.658, with a 95%CI ranging from 0.354 to 0.819. This moderate ICC value suggests that there is some variability in the results obtained by the two examiners, but it is still within an acceptable range.

The study also calculated the SEM and MDC values, which are important indicators of the precision and reliability of the measurement method. The SEM value obtained was 3.50°, and the MDC value obtained was 9.70° (Table 2).

### 3.3. Concurrent Validity

**Figure 3:**
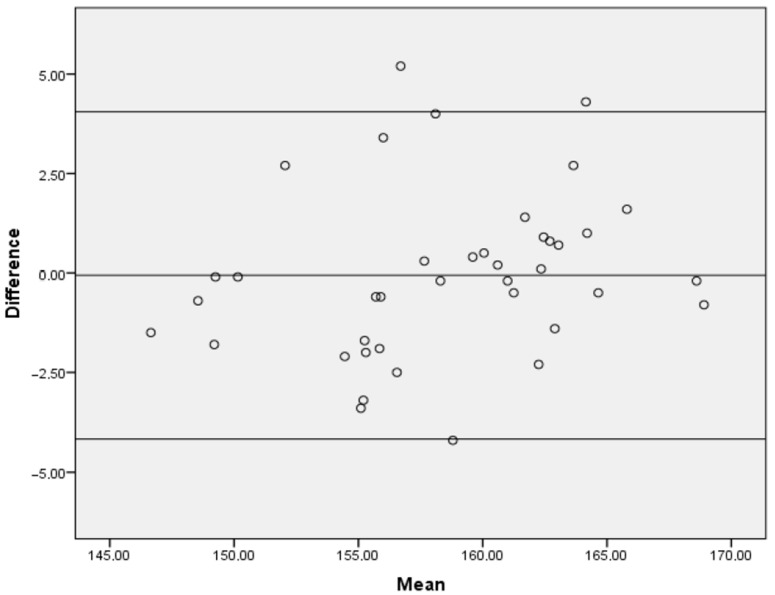
Bland–Altman plot.

The 2D analysis of the data collected using Kinovea software yielded a mean value of 158.48 ± 5.75. To assess the concurrent validity of this measurement method, the study calculated the correlation between the beta application based on computer vision and Kinovea. The results showed a strong correlation between the analysis of the knee valgus angle measured by the beta application based on computer vision and the Kinovea software (r = 0.931; *p* < 0.001). The SEM value, which represents the standard error of measurement between the two instruments, was found to be 1.44°. This indicates a high degree of precision and consistency between the two measurement methods. To further illustrate the agreement between the two instruments to measure the knee valgus angle, the study presented a Bland–Altman plot in Figure 3.

### 3.4. Time Spent on Each Measurement

The study also examined the time required for conducting measurements using the two different methods. Specifically, the time spent on the measurement of the 42 photos using Kinovea software was found to be 943 s, whereas the mean time required for conducting measurements using the beta application based on computer vision was 133 s.

## 4. Discussion

The present study investigated the test-retest and inter-rater reliability and the concurrent validity of a beta application based on computer vision for the measurement of the knee valgus angle in elite handball athletes. It was found that the test-retest reliability was excellent for both examiners, and the inter-rater reliability was moderate. A very strong relationship and agreement between the beta application and the Kinovea software for measuring the knee valgus angle in elite handball athletes was found. The time spent on calculating the knee valgus angle was higher in Kinovea software than in beta application based.

This study yielded promising results, indicating that a beta application is a valid alternative to the traditional 2D analysis for evaluating knee valgus angle in the frontal plane. The knee valgus angle SEM value obtained between the beta application based on computer vision and the Kinovea software was 1.44°, which suggests a high level of agreement between the two methods. While it would be ideal to compare the beta application based on computer vision with a 3D analysis system such as the ©VICON Motion System from Oxford, UK, the similarity of the results with previous studies that have compared traditional 2D analysis with 3D analysis systems (which reported SEM values ranging from 2° to 5°) is encouraging [32]. The findings of the present study also showed that the knee valgus angle values observed in elite handball athletes during a single-leg squat were lower than those reported in other studies with elite handball athletes [10]. However, it is important to note that the study did not focus on describing the knee valgus or its relationship with injury risk. Rather, the primary objective was to assess the reliability and validity of the beta application in measuring the knee valgus angle.

The quantification of the knee valgus angle has been identified as a significant indicator in the prevention of knee injuries, as supported by research findings [33]. Moreover, it has been reported that the quantification of the knee valgus angle is a more reliable and valid measure as compared to visual assessment [34]. Consequently, it is crucial to equip clinicians with dependable instruments that enable them to objectively evaluate clinical signs and symptoms. Additionally, these tools should not consume excessive time, which could otherwise be used to administer appropriate treatment for each pathology. The current study indicates that the time spent on the knee valgus angle assessment using Kinovea software was longer compared to the time spent on the beta application based on computer vision. Interestingly, the beta application based on computer vision demonstrated lower SEM than the two-dimensional (2D) analysis of the frontal-plane knee valgus [35]. Furthermore, the MCD observed in our study was akin to that reported by Carvalho et al. in professional volleyball athletes, but in our case, during landing after a jump [36]. There is potential for the beta application based on computer vision to evolve. Specifically, an application has been proposed where markers need to be manually placed on photos, and clinicians need to invest time in this task. However, it is possible to improve this application by programming it as guidelines, wherein the computer vision system could autonomously search for the ASIS, the midpoint of the patella, and the anterior tibial tuberosity. This could streamline the assessment process and further enhance the clinical utility of the beta application based on computer vision [22].

The findings of this study have significant implications for clinical practice, highlighting the potential benefits of employing computer vision-based tools for measuring knee valgus angles in elite handball athletes. In settings where 3D systems are not available, the use of computer vision applications can provide a reliable and valid alternative [19]. It seems that technological advances could help make the measurement of joint angles faster and more reliable, and there is also a trend that markers are no longer needed [20,22]. By harnessing the capabilities of computer vision applications and machine learning, clinicians could gain valuable insights into the risk factors associated with different activities or the elderly, facilitating the development of targeted injury prevention strategies [21,37]. Additionally, objective measures of changes in joint health over time could be obtained, enabling the evaluation of training and rehabilitation programs. With the growing emphasis on evidence-based practice in healthcare, the use of objective, quantifiable measures provided by computer vision-based applications could enhance the accuracy of clinical decision-making, ultimately leading to improved patient outcomes [19,21,38].

It is important to acknowledge several limitations in our study. Firstly, during the study, we only measured the knee valgus angle and did not measure the hip and ankle frontal plane. This narrow focus may not provide a complete picture of the biomechanical factors contributing to knee injuries in handball athletes. Secondly, the sample size was limited to male elite handball athletes, which may limit the generalizability of findings to other athletes or pathologies. Future studies should aim to replicate our results with larger, more diverse samples that include both male and female athletes across a range of skill levels and sports. Moreover, the current study only evaluated the reliability and validity of the beta application based on computer vision over a short period of time. Future studies should explore the long-term reliability and reproducibility of the method to ensure that it can be used effectively in clinical practice. In addition, once the reliability and validity of a method for a specific physical examination are established, it is essential to analyze its ease of use and cost-effectiveness to ensure that it can be implemented in routine clinical practice. Despite these limitations, our study contributes to the growing body of literature supporting the use of computer vision-based applications in the assessment of the knee valgus angle. Further research in this area is warranted to fully understand the potential benefits of this innovative technology in clinical practice.

## 5. Conclusions

The present study demonstrated that the use of a beta application based on computer vision for measuring knee valgus angle in elite handball athletes has yielded promising results in terms of reliability and validity. Specifically, our findings indicate that the beta application based on computer vision exhibited excellent test-retest reliability and moderate inter-rater reliability, suggesting that it can be consistently used by clinicians in the assessment of knee valgus angle. Furthermore, the strong agreement between the beta application based on computer vision and the 2D Kinovea software suggests that the beta application based on computer vision can provide an effective alternative to traditional 2D systems for assessing knee valgus angles in handball athletes.

These results provide further evidence of the potential benefits of incorporating computer vision technology into clinical practice. As this technology continues to evolve and improve, it has the potential to revolutionize the way clinicians measure and assess joint function in athletes and other patient populations. The high level of reliability and validity demonstrated by the beta application based on computer vision in this study supports its use as a valuable tool for the objective evaluation of knee valgus angles in elite handball athletes and highlights its potential for use in other clinical contexts.

However, the results must be interpreted with caution. The beta application based on computer vision was only utilized for the measurement of the knee valgus angle, and therefore, the findings cannot be extrapolated to other knee angles. Additionally, the generalizability of the results to female athletes is limited since the sample in our study consisted exclusively of male athletes.

## Figures and Tables

**Table 1:** Test-retest reliability of the beta application based on computer vision.

	Assessment 1 (M ± SD)	Assessment 2 (M ± SD)	ICC	CI 95%	SEM	MDC	CV
Examiner 1	158.54 ± 5.27°	156.52 ± 8.76°	0.859	0.734–0.926	2.63°	7.28°	3.34%
Examiner 2	154.42 ± 6.72°	153.34 ± 6.38°	0.933	0.874–0.956	1.69°	4.68°	4.28%

M: mean; SD: standard deviation; ICC: intraclass correlation coefficient; CI: confidence interval; SEM: standard error measurement; MDC: minimal detectable change; CV: coefficient of variation.

**Table 2:** Inter-rater reliability of the beta application based on computer vision.

Examiner 1 (M ± SD)	Examiner 2 (M ± SD)	ICC	CI 95%	SEM	MDC
158.54 ± 5.27°	154.42 ± 6.72°	0.658	0.354–0.819	3.50°	9.70°

M: mean; SD: standard deviation; ICC: intraclass correlation coefficient; CI: confidence interval; SEM: standard error measurement; MDC: minimal detectable change.

## Data Availability

The data analyzed in this study are included in this published article. The dataset is available from the corresponding author upon reasonable request.
